# A COG in the machine: how good Golgi makes good pollen

**DOI:** 10.1093/plphys/kiab078

**Published:** 2021-02-28

**Authors:** Elisa Dell’Aglio

**Affiliations:** Institut National des Sciences Appliquées de Lyon, 69100 Villeurbanne, France

Pollen, the plant male gametophyte, is a multinucleated structure that must develop into a pollen tube to penetrate through the style of the pistil and fertilize the female gametophyte. The development of the pollen tube is regulated by a molecular dialogue mediated by the ovary through the female pistil (Higashiyama and Takeuchi, 2015). Pollen tube extension speeds can reach 3 µm per second, making it the fastest growing plant cell known (Selinski and Scheibe, 2014).

Pollen tube extension proceeds by exocytotic activity of Golgi-derived vesicles at the pollen tip, resulting in a polarized tube membrane extension (Cai et al., 2015). These vesicles transport proteins and lipids to be inserted in the apical plasma membrane, as well as polysaccharide-synthesizing enzymes (such as callose synthase) and pectins that will build the primary cell wall (Cai et al., 2015). The essential role of Golgi vesicle trafficking has been revealed by the severe phenotypes of mutant affected in cytoskeletal proteins that guide vesicle movements (Xu and Huang, 2020) and of mutants affected in proteins involved in vesicle trafficking and fusion to their target membranes (Zhou et al., 2020; Lund et al., 2020). Among the latter, the conserved oligomeric Golgi (COG) complex is an eight-subunit heterocomplex essential for orchestrating vesicle movements from the Golgi to the endoplasmic reticulum (ER). This process, called retrograde transport, is essential for recycling membranes and proteins, such as glycosyltransferases (Cai et al., 2015). Mutations in COG components stall pollen tube elongation (Tan et al., 2016), revealing correct retrograde transport ensures the correct functionality of the Golgi apparatus by counterbalancing the opposite vesicle movement from the ER to the Golgi (the so-called anterograde trafficking).

In this issue of *Plant Physiology*, Rui and colleagues (2021) show the COG complex interacts with two SYP3 (SYntaxin of Plants family 3) proteins, called SYP31 and SYP32. The interactions appear to be mediated by COG subunit 3, which bound SYP31 and SYP32 in pull-down assays both *in vivo* and *in vitro*, as well as in yeast two-hybrid and by split luciferase assays. Moreover, the absence of SYP31 and SYP32 disrupted COG association with the Golgi apparatus and led to its diffuse localization into the cytoplasm, thus showing SYP3s are essential for addressing the COG complex to the Golgi.

The Arabidopsis (*Arabidopsis thaliana*) proteins SYP31 and SYP32 belong to the SNARE (Soluble Nsf-Associated Protein REceptor) protein family, and therefore likely mediate the fusion of vesicles to their target membrane (Wang et al., 2017). This, together with previous evidence indicating *SYP31* overexpression inhibits anterograde trafficking in tobacco (*Nicotiana tabacum*) protoplasts (Bubeck et al., 2008), led the authors to hypothesize a role for SYP31 in ER-Golgi trafficking. Moreover, since SYP31 and SYP32 are the only two Golgi-localized SNARE proteins belonging to the Qa subfamily in Arabidopsis, their function might be redundant.

To assess the importance of SYP31 and SYP32 for pollen viability and development, the authors self-pollinated one heterozygous *syp31* and two heterozygous *syp32* T-DNA insertion lines and then established crosses with wild-type Col-0 plants. The results of these crosses showed SYP31 is not essential for correct pollen development, while SYP32 mutations led to reduced pollen formation (only 36% or 41% of the offspring contained the pollen-transmitted mutation, respectively, when mutants were crossed with wild-type female gametophytes). However, it was not possible to obtain double mutants after crossing the heterozygous *syp31* line with any heterozygous *syp32* line. This, together with the rescue of the mutant phenotype with *SYP31*- or *SYP32*-overexpression, confirmed that SYP31 and SYP32 are redundant and that their function is essential for pollen development.

A follow-up on pollen grain maturation revealed pollen degeneration, including DNA degradation, in *syp31 syp32* double mutants as early as after the first mitotic division (bicellular pollen stage), when *SYP31* and *SYP32* expression begins. Interestingly, these severe phenotypes appeared much earlier than the abnormal phenotype of the *cog* mutant, which is restricted to pollen tube growth (Tan et al., 2016). This suggested SYP31 and SYP32 roles are not only related to COG-dependent retrograde signaling, but also to many other aspects of Golgi vesicle trafficking.

**Figure 1:**
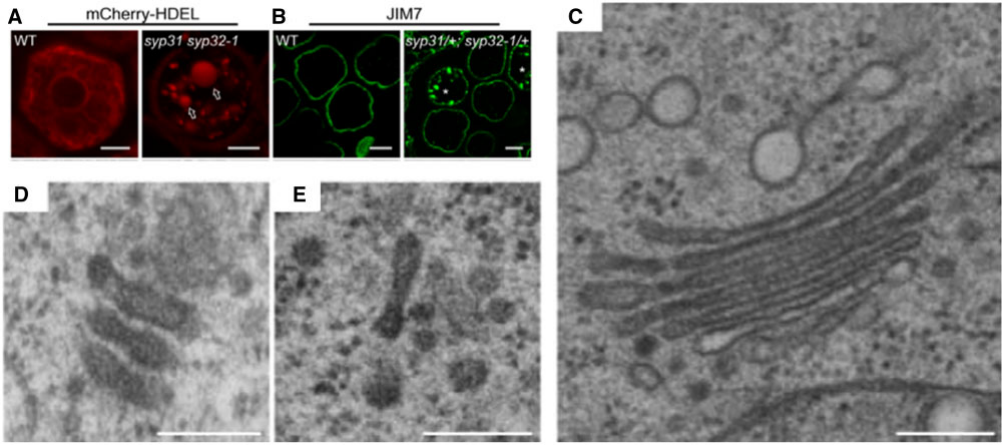
Defective morphology and membrane trafficking in *syp31 syp32* pollen grains. A–B, Confocal images of wild-type and *syp31 syp32* pollen. Bars = 5 µm. A, Localization of overexpressed mCherry-HDEL protein (red signal). B, Localization of pectins labeled with the JIM7 antibody (green signal). In the double mutant pollen grains, the mCherry-HDEL mislocalized to the vacuole (arrows) instead of the ER, and pectins accumulated in punctuated structures in the cytoplasm instead of being addressed to the plasma membrane as in the wild type (asterisks). C–E, TEM images of wild-type (C) or *syp31 syp32* (D–E) bicellular pollen, showing the morphology of the Golgi apparatus. Bars = 200 nm (adapted from Rui et al., 2021).

In agreement with this hypothesis, Rui and colleagues observed a relocation of ER proteins in the vacuole, as well as cytoplasmic pectin accumulation points in *syp31 syp32* double mutants, which indicate deficient vesicle trafficking, both to the ER and to the plasma membrane (Figure 1, A and B). Moreover, the cellulose layer around the *syp31 syp32* double mutant pollen grains was unevenly distributed, with empty patches on the pollen grain surface, suggesting defects in cellulose and cell-wall related enzymes trafficking to the plasma membrane. The morphology of the Golgi apparatus was also deeply affected (Figure 1, C–E), with on average half the number of cisternae and half the length of wild-type plant cells. These results demonstrate disrupting SYP3s affects Golgi morphology and functionality, for both retrograde and anterograde trafficking.

In conclusion, the Qa-type SNARE proteins SYP31 and SYP32 are essential for ensuring correct membrane transport in Arabidopsis pollen grains. Their functions are partly due to their role in mediating COG interaction with the Golgi and therefore for correct anterograde vesicle trafficking. However, SYP3s seem to orchestrate both anterograde and retrograde vesicle trafficking, likely by interacting with many other vesicle trafficking signaling components which remain to be identified.
